# Cranio-Facial Characteristics in Autism Spectrum Disorder: A Scoping Review

**DOI:** 10.3390/jcm13030729

**Published:** 2024-01-26

**Authors:** Giuseppe Quatrosi, Dario Genovese, Giuseppe Galliano, Hugo Zoppé, Emanuele Amodio, Fréderique Bonnet-Brilhault, Gabriele Tripi

**Affiliations:** 1 Department of Psychology, Educational Science and Human Movement, University of Palermo, 90128 Palermo, Italy; giuseppe.quatrosi01@unipa.it; 2 Department of Health Promotion, Mother and Child Care, Internal Medicine and Medical Specialties (PROMISE), University of Palermo, Via del Vespro, 133, 90127 Palermo, Italy; giuseppe.galliano@community.unipa.it (G.G.); emanuele.amodio@unipa.it (E.A.); gabriele.tripi@unipa.it (G.T.); 3 UMR 1253 iBrain, Inserm, Université de Tours, 37020 Tours, France; hugo.zoppe@univ-tours.fr (H.Z.); frederique.brilhault@univ-tours.fr (F.B.-B.); 4 Excellence Center for Autism and Neurodevelopmental Disorders, CHRU de Tours, 37000 Tours, France; 5 Department of Child and Adolescent Psychiatry, EPSM du Loiret/Centre Hospitalier Universitaire d’Orléans, Université d’Orléans, 45100 Orléans, France

**Keywords:** spectrum disorders, morphology, neurodevelopment, cranio-facial characteristics

## Abstract

Autism spectrum disorders (ASD) consist of a complex group of neurodevelopmental disorders characterised by qualitative impairments of social interactions, communication abilities, and a limited, stereotyped, and repetitive selection of interests and activities. In light of the imperative to identify a possible biomarker for ASD, it has been determined that craniofacial anomalies serve as significant risk factors for neurodevelopmental disorders. The aim of this scoping review is to deepen the knowledge of the scientific literature related to cranio-facial characteristics in individuals with ASD, with a particular focus on recent research advancements. The review was performed by employing the search strings ((“*Autism Spectrum Disorder*” *OR autism OR ASD OR* “*Autism Spectrum*”*) AND (*“*facial morphology*” *OR* “*facial phenotype*”)) on the databases PubMed/MEDLINE, Scopus, and ERIC as of March 9, 2023. The review comprised seven studies whose findings were obtained through quantitative analysis of Euclidean distances between anatomical landmarks. The examination of facial abnormalities represents a possible reliable diagnostic biomarker that could aid in the timely identification of ASD. Phenotypic characteristics that may serve as predictive indicators of the severity of autistic symptoms can be observed in certain individuals with ASD by applying anthropometric and instrumental measurements. The presence of a phenotype characterised by an increased intercanthal distance and a reduced facial midline height appears to be associated with a higher degree of severity in autistic symptoms. In addition, it is worth noting that facial asymmetry and facial masculinity can be considered reliable indicators for predicting a more severe manifestation of symptoms.

## 1. Introduction

Autism spectrum disorders (ASD) encompass a complex group of neurodevelopmental disorders (NDD) [1]. These disorders are distinguished by qualitative impairments of social interactions and communication and a limited, stereotyped, and repetitive range of interests and activities [2].

The aetiology of ASD is believed to depend on an intricate interaction between endogenous and exogenous factors, which could be embodied in a multifactorial framework involving genetics, environmental, and epigenetic factors that collectively contribute to atypical brain development [3,4]. 

To date, the identification of valid and specific biomarkers for the diagnosis of ASD remains unresolved. The examination of the facial phenotype in individuals with ASD holds a significant opportunity for advancing our understanding of this condition. By exploring the cranio-facial characteristics, researchers aim to uncover underlying neurobiological mechanisms that contribute to the pathology, other than to improve the accuracy of diagnostic procedures for ASD [5].

Over the past two decades, the application of neuroimaging techniques has contributed enormously to the study of pathological changes occurring within the brain of individuals with ASD in vivo. The observed changes appear to be accompanied by atypical trajectories of brain maturation, resulting in neuroanatomical, functional, and connectivity differences that are presumably associated with the expression of autistic symptoms and traits. Children with ASD exhibit a distinct trajectory of brain development characterised by an initial phase of overgrowth, followed by arrested growth and a potential decrease in brain volume at older ages. Furthermore, the aforementioned differences indicate different patterns across different brain regions, wherein the frontal and temporal regions display a higher degree of involvement when compared with the parietal and occipital lobes [6]. Moreover, it has been observed that children with ASD tend to have significant alterations in the maturation of the social brain. These modifications manifest as pathological changes in the fronto-temporo-parietal cortex, amygdala, and cerebellum [7,8]. 

In light of these findings and in response to the need to identify a possible biomarker for ASD, it has been established that cranio-facial anomalies show characteristics that pose as risk factors for neurodevelopmental disorders [9]. Indeed, these could represent physical indicators of embryonic development that could potentially contribute to the occurrence of complications during early stages, even pre-conceptional, phases of ontogeny in autism spectrum disorders (ASD) [10].

The scientific literature suggests that ASD may have its origins during the prenatal period [11]. This hypothesis is supported by evidence indicating that the developing central nervous system (CNS) is particularly susceptible and vulnerable to external factors during this critical phase [12,13]. Furthermore, it has been proposed that the prefrontal cortex may be influenced by these prenatal insults during mid-foetal development [14].

The primary aim of this review is to deepen the knowledge of the scientific literature, including the most recent advances in research, on cranio-facial characteristics in individuals with ASD.

## 2. Materials and Methods

For this scoping review, the research group employed the Preferred Reporting Items for Systematic Reviews and Meta-Analyses extension for scoping reviews (PRISMA-ScR) criteria to accurately document the methodology and results of the study [15].

### 2.1. Research Questions

The authors aimed to address any concerns arising from the gaps identified in the aforementioned area of study. In particular:

1. Is it possible to identify a facial phenotype associated with ASD?
2. How does facial phenotype contribute to our understanding of brain development in ASD?
3. Could cranio-facial characteristics predict behavioural features in ASD individuals?

### 2.2. Search Strategy

In conducting this scoping review, three peer-reviewed literature database sources were consulted: PubMed/MEDLINE, Scopus, and ERIC. The literature review search was conducted by combining free-text terms and medical subject headings (MeSH) on 9 March 2023. The search strategy consisted of both ASD-specific and face characteristics-specific phrases. Subsequently, each term was connected using the Boolean operators “AND” and “OR”, resulting in the following search string:

*((“Autism Spectrum Disorder” OR autism OR ASD OR “Autism Spectrum”) AND (“facial morphology” OR “facial phenotype”))*

### 2.3. Study Selection

The search query enabled the authors to identify a total of 553 research papers, which were then reduced to 518 after eliminating any duplicates. Mendeley version 1.13 facilitated the removal of duplicates. 

The inclusion criteria—implemented during both the screening and eligibility phases—were specifically developed to identify peer-reviewed studies assessing the facial features of individuals diagnosed with essential ASD. During the screening phase, the inclusion criteria were used to assess the title and abstract of each identified article. Subsequently, during the eligibility phase, the inclusion criteria were applied to the full texts of the articles that successfully completed the screening.

The researchers excluded studies that did not report or employ either facial traits or ASD-specific information. The scoping review excluded papers that were not written in English, as well as reviews, notes, book chapters, conference papers, short surveys, case reports, and studies that applied a qualitative research approach.

Inclusion criteria:

- Original articles.
- Studies evaluating cranio-facial characteristics in individuals with ASD.
- Clinical evaluation of individuals with ASD.
- Methodological assessment of cranio-facial characteristics.
- Written in the English language.

Exclusion criteria:

- Studies that did not analyse cranio-facial features.
- Studies that were not conducted on individuals with ASD.
- No clinical examination of the participants was conducted.
- Cranio-facial features are not assessed.
- Not written in the English language.
- Specific research on genetic disorders or mutations.
- Studies that focused specifically on prenatal alcohol exposure.
- Short surveys, reviews, book chapters, notes, and conference papers.

### 2.4. Charting the Data

The researchers employed a “descriptive analytical” approach to gather all relevant data [16]. Specifically, each article provided the following data: (1) the article’s first author and pertaining citation; (2) the article’s title; (3) publication year; (4) the country where the research was conducted; (5) design of the research; (6) objectives of the study; (7) participants’ number; (8) age range; (9) ethnicity; (10) matching; (11) evaluation tools for autism; (12) exclusion of distinct syndromes; (13) methods and measurement; (14) evaluated areas and (15) main results.

Using the Quality Assessment for Diverse Studies (QuADS) tool, a revised version of the Quality Assessment Tool for Studies with Diverse Design tool, the quality of each identified article was appraised [17]. Each criterion is rated on a 4-point scale ranging from 0 to 3, with 0 representing no mention of the criterion, 1 representing a general mention/description, 2 representing a good description/analysis, and 3 representing a thorough analysis of the item. Accordingly, the total score for each article ranged from 0 to 39; the higher the score, the higher the quality of the article.

### 2.5. Collating, Summarising, and Reporting the Results

The findings are presented in Section 3. The items previously mentioned in the “Charting the data” section were used as a technique to collect and summarise the data.

## 3. Results

### 3.1. Literature Search

**Figure 1:**
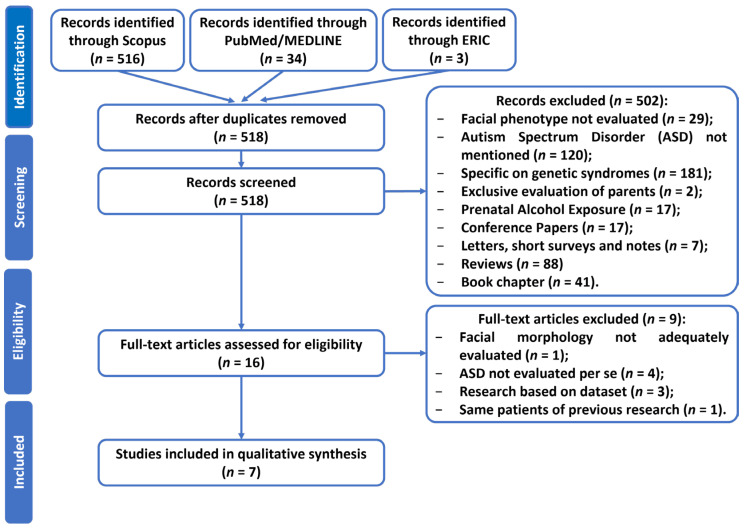
Flowchart representing PRISMA flow diagram of studies’ screening and selection.

Figure 1 displays the results of the PRISMA-ScR phases.

As per the identification phase, an overall total of 553 records were retrieved from the Scopus (516), PubMed/MEDLINE (34), and ERIC (3) searching platforms. After removing thirty-five duplicate records, the remaining 518 records were included for further examination.

After the screening process, a total of 502 records were excluded due to the following reasons: 181 records (36.1%) focused on the exploration of specific genetic disorders or mutations associated with ASD; 120 reports (23.9%) did not make any mention of ASD. A smaller subset of studies, specifically 29 (5.8%), did not aim to explore facial phenotypes. Furthermore, 17 publications (3.4%) solely assessed prenatal alcohol exposure. Only two reports (0.3%) exclusively conducted an evaluation of parents. Out of the remaining 153 records, a total of 88 (17.5%) were reviews, 17 (3.4%) were conference papers, 7 (1.4%) were letters, short surveys, and notes, and 41 (8.2%) were book chapters.

A total of sixteen full-text papers were assessed for eligibility, out of which nine were excluded based on the following rationales: Four studies did not specifically analyse ASD itself, accounting for 44.5% of the total. Three studies relied on datasets consisting of photographs, representing 33.3% of the total. One study did not adequately assess facial morphology, making up 11.1% of the total. Finally, one study examined the same group of patients as a previous study discussed in this article, accounting for 11.1% of the total.

Seven studies were included in the scoping review after the eligibility step was completed [18,19,20,21,22,23,24].

### 3.2. Characteristics of the Included Studies

The characteristics of the seven manuscripts included by the researchers are summarised in Table 1. Overall, 6/7 reports [18,19,20,21,22,23] applied a case–control study design, whereas the remaining one [24] opted for a cross-sectional study.

Out of the seven articles reviewed, one article (14%) was conducted in France, Europe [24]. Two reports (29%) were performed in Australia [19,22]. Three articles (43%) were directed in North America, with two articles [18,21] in the United States and one record [20] in Canada; the remaining article (14%) was conducted in Turkey [23].

The QuADS scores of each item were assessed, resulting in a range of 41.0% to 87.2% and a mean score of 69.2%. Most of the articles received lower ratings for the following criteria: theoretical or conceptual underpinning to the research; statement of research aim(s); a clear description of the research setting and target population; properness of the sampling to address the research aim(s); provision of the recruitment data. At the same time, reports had higher ratings in the following items: properness of the study design to address the stated research aim(s); rationale for the choice of data collection tool(s); properness of format and content of data collection tool to address the stated research aim(s); description of data collection procedure; justification for the analytic method selected; evidence that the research stakeholders have been considered in research design or conduct; critical assessment of strengths and limitations.

Each of the articles was published after 2000.

### 3.3. Evaluation of Cranio-Facial Characteristics in ASD Individuals

The information obtained from each publication is shown in Table 2.

In six out of seven studies (86%), researchers examined the cranio-facial characteristics of individuals with ASD in comparison to those without the condition [18,19,20,21,22,23]. The remaining study (14%) focused on assessing the cranio-facial features of a group of autistic boys and girls without including a control group [24].

With regard to the age group composition of the sample under investigation, six research papers (86%) focused on evaluating the cranio-facial features of individuals in the prepubertal stage [19,20,21,22,23,24]. In contrast, only one article (14%) included adolescent participants, ranging up to 18 years of age [18].

With reference to gender, it is worth noting that in 43% (3 out of 7) of the examined studies, only a male population was included [18,20,21], while in the remaining 57% (4 out of 7) of the studies, also the female population was considered, albeit with smaller sample sizes compared to their male counterparts [19,22,23,24].

In almost all of the articles (86%), the focus was solely on individuals of Caucasian ethnicity [18,19,20,21,22,24], while in the study conducted by Topal et al. [23] in Turkey, accounting for 14% of the articles, the ethnicity of the participants was not explicitly stated.

In 5/7 studies (71%), participants who presented identifiable genetic syndromes or had known specific genetic mutations were excluded [18,19,20,21,24]. Conversely, in the remaining 29% of studies, no specific criteria were outlined for the exclusion of participants with genetic syndromes or mutations [22,23].

In a study conducted by Boutrus et al. [19], individuals with ASD were examined in relation to both a control group of typically developing individuals and their non-autistic siblings.

Each of the studies included in this review conducted a clinical examination for the diagnosis of ASD in accordance with the Diagnostic and Statistical Manual of Mental Disorders (DSM) criteria. In almost all the reports (86%), the inclusion of additional diagnostic tests for ASD was documented [18,19,20,21,22,24]. The Autism Diagnostic Interview-Revised (ADI-R) [25] and the Autism Diagnostic Observation Schedule (ADOS) [26] were employed in 3 (43%) out of 7 articles [18,20,21]. In two out of seven studies (29%), only the ADOS was adopted as the assessment tool [19,22]. In one study (14%), the ADI-R and the Childhood Autism Rating Scale (CARS) [27] were both used to diagnose individuals with ASD together with the Weschler Abbreviated Intelligence Scale-III (WAIS III) [28], a useful assessment tool to evaluate the intellectual quotient (IQ) of patients diagnosed with ASD [24]. No use of psychodiagnostic tests to support the diagnosis of ASD was documented in the study of Topal et al. (14%) [23].

All studies included in this review assessed the cranio-facial features of individuals with ASD. In 5/7 studies (71%) [18,19,20,21,22], the cranio-facial characteristics of the participants were examined using 3D imaging techniques and the 3DMD System software (3DMD LLC, Atlanta, GA, USA). In the remaining two studies (29%), the cranio-facial characteristics of the participants were evaluated using photogrammetry. Specifically, Tripi et al. [24] employed a combination of classical anthropometry and photo analysis with FlashCAD software (FlashCAD^®^ Digitarch srl, Rome, Italy), while Topal et al. [23] applied ImageJ software (Version 1.50b, National Institutes of Health, Bethesda, MD, USA) for photo analysis. Considering the broad diversity of tools employed in the analysis of the seven studies under review, as well as the different indices examined in the studies in which the 3DMD software was employed, it was considered appropriate to provide a separate description for every report individually.

Hammond et al. [20] aimed to examine and compare the facial morphology of boys with ASD and their first-degree relatives with that of unrelated control individuals. The researchers used the 3DMD system to determine the asymmetry index for numerous areas, including the face, supra-orbit, periorbita, perinasal, and perioral regions. They discovered that the facial features of boys with ASD exhibited an unusual pattern of right-sided dominance in the supraorbital and periorbital regions located anterior to the frontal cerebral pole.

The study conducted by Aldridge et al. [18] sought to examine whether children diagnosed with ASD exhibited a subtle yet discernible facial phenotype in comparison to TD children. Additionally, the researchers aimed to determine if there were subcategories of facial phenotypes within the ASD group. The cranio-facial characteristics of the population part of the study were examined using the 3DMD system. Afterwards, subgroups were identified by using the Euclidean Distance Matrix Analysis and the Principal Coordinates Analysis. Statistically significant differences were observed in facial morphology when comparing children with ASD to typically developing individuals. These differences included a notable increase in the breadth of the mouth, orbits, and upper face, along with a flattened nasal bridge and decreased height of the philtrum and maxillary region. Furthermore, the study identified two separate subgroups presenting distinct clinical characteristics. One subgroup, referred to as subgroup 1, exhibited a reduced height of the facial midline and increased breadth of the mouth, as well as an increased length and height of the chin. Another subgroup, identified as subgroup 2, displayed an increased breadth of the upper face along with a decreased height of the philtrum. The subgroups exhibited distinct biological and etiological characteristics. Subgroup 1 exhibited a greater severity of ASD and revealed higher Social Communication Questionnaire (SCQ) lifetime scores compared to subgroup 2. Moreover, subgroup 1 displayed lower levels of IQ. Subgroup 1 exhibited multiple features that are indicative of an unfavourable outcome, such as a heightened susceptibility to seizures and a higher occurrence of language regression at the onset of ASD. Subgroup 2 exhibited more conformity with the diagnostic criteria associated with Asperger syndrome.

The objective of the study conducted by Obafemi-Ajayi et al. [21] was to investigate the potential of facial morphology as a reliable biomarker for distinguishing distinct forms of ASD. The clusters were identified through the analysis of measurements obtained from the 3DMD system, specifically concerning facial height, mid-face height, mid-face breadth, lower face height, and mouth width. The group of researchers discovered that it was possible to discern three distinct clusters. Cluster 1 was characterised by an overall decrease in surface facial heights, coupled with a wider maxillary midface from the temporal landmark to the lower nose landmarks. Cluster 2 exhibited a general expansion in surface facial heights, a reduction in mid-face height, and the broadest mouth widths. Cluster 3 showed characteristics located between those observed in cluster 1 and cluster 2. The results indicated that cluster 2 exhibited severe ADI-R scores, low cognitive and functional IQ scores, the highest maternal SRS scores, and significant language regression. These findings align with the subgroup 1 identified by Aldridge et al. [18].

Tan et al. [22] aimed to examine the potential presence of heightened facial masculinity in prepubescent boys and girls diagnosed with ASD in comparison to TD controls. The 3DMD System software was used to analyse a range of facial variables, including facial area, gender scores, alar-base width, nose height, upper lip height, outer-canthal width, forehead height, and nose height. It was observed that the ASD group exhibited higher facial masculinity compared to the control group, for both sexes. Additionally, there was a correlation observed between facial masculinity in individuals with ASD and greater difficulties in social communication, as measured by the Social Affect score derived from the ADOS-G.

In their study, Tripi et al. [24] sought to examine the topographical pattern of craniofacial abnormalities in children with ASD and determine if these abnormalities were associated with the severity of ASD symptoms and overall functioning. The determination of different cranio-facial indexes, including the cephalix index, facial index, intercanthal index, nasal index, and facial-mouth width index, occurred using a combined approach involving classical anthropometry and photogrammetry. The study revealed several key findings, one of which was the identification of two cranio-facial markers that exhibited a significant correlation with the severity of ASD. These markers were in particular characterised by an increase in orbital hypertelorism and a decrease in the height of the facial midline.

Boutrus et al. [19] aimed to examine the reproducibility of increased facial asymmetry in children with ASD in comparison to their non-autistic counterparts. The 3DMD System software was used to analyse cranio-facial features and evaluate three specific types of asymmetries: horizontal, vertical, and depth facial asymmetry. The study revealed that individuals with ASD exhibited greater depth-wise facial asymmetry (FA) in comparison to age-matched TD children and their full siblings who did not have ASD. Furthermore, a significant association was observed between the severity of ASD symptoms and FA.

The study conducted by Topal et al. [23] attempted to investigate the orbital region in children diagnosed with ASD and compare it to a control group. This investigation was carried out using the method of photogrammetry. The researchers conducted measurements on various facial parameters, including the outer canthal distance, palpebral fissure distance (also known as intercanthal distance), bilateral inner canthal distances, and interpupillary distance. The findings of this study indicate that there was a significant increase in orbital distances among males with ASD, while no significant difference was observed in females with autism when compared to the control group.

## 4. Discussion

### 4.1. Is It Possible to Identify a Facial Phenotype Associated with ASD?

Despite the limited number of studies employing quantitative anthropometric methodologies to assess cranio-facial characteristics in individuals with ASD and the discrepancies in methodologies employed, the reviewed studies provide evidence that suggests the existence of potentially significant facial phenotypic patterns that can be observed in certain individuals with ASD. Furthermore, it is relevant to note that all the studies included in this review obtained their results from the quantitative analysis of Euclidean distances between anatomical landmarks.

#### 4.1.1. Increased Intercanthal Distance and Decreased Height of the Facial Midline

This review reveals a prevalent characteristic observed within the autistic population, namely an elevated intercanthal distance.

In their study, Aldridge et al. [18] observed a correlation between an increase in orbital distance and several cranio-facial changes, including an increase in the breadth of the mouth and the upface. Additionally, they observed a flattened nasal bridge and a reduction in the height of both the philtrum and the maxillary region. The results of Obafemi-Ajayi et al. [21] are in line with and support the previously mentioned results, as they were conducted on a partially overlapping dataset (52 out of the sixty-three participants used in the previous study, with an additional 10 male participants). However, Obafemi-Ajayi et al. employed a distinct research methodology. Tan et al. [22] examined various facial variables in an attempt to identify a discernible pattern of facial masculinity in individuals with ASD; the findings of this study indicate an observed increase in outer-canthal distance. Tripi et al. [24] carried out an investigation within the autistic population to explore potential markers associated with the severity of autism. Notably, the study reported the presence of two potential markers: hypertelorism and a reduction in the height of the facial midline. It is worth noting that the study did not include a control group. Finally, in the study conducted by Topal et al. [23], it was observed that the interorbital distance exhibited an increase in the group of boys diagnosed with ASD. However, no statistically significant differences were identified between the ASD girl group and the control group in terms of interorbital distance.

An increase in the intercanthal distance, also known as mild hypertelorism, may serve as a potential supplementary biomarker that might help in the prompt identification of individuals with ASD while also contributing to a better comprehension of the neurodevelopmental mechanisms associated with this condition (see Section 4.3). At the same time, it is important to bear in mind that this trait is observed solely in a subset, albeit a substantial one, of individuals with ASD. Consequently, it cannot serve as a standalone biomarker for diagnostic purposes. It is important to acknowledge that the anthropometric modifications observed in these studies have been assessed using sophisticated techniques for the examination of cranio-facial characteristics, which are not readily reproducible in an outpatient setting. Furthermore, these modifications may not always be discernible to the unaided eye. In this review, all studies that examined specific syndromes or genetic mutations, as well as those that focused on individuals who were prenatally exposed to alcohol, were excluded. Consequently, it is important to emphasise that the data discussed in this review are more suitable for conditions similar to essential autism than syndromic autism. Syndromic autism, characterised by specific etiopathogenetic and neurodevelopmental mechanisms, may exhibit distinct cranio-facial abnormalities.

Considering the aforementioned limitations, it is plausible to consider the measurement of intercanthal distance as a potentially effective early biomarker in the clinical context, provided that it is supported by a meticulous clinical evaluation of the child. Furthermore, in accordance with the previously mentioned studies, it is plausible to consider it as a potential predictive marker of the greater severity of symptoms associated with ASD (see Section 4.2).

#### 4.1.2. Facial Asymmetry (FA)

Facial asymmetry (FA) is another factor that has been examined within the framework of cranio-facial characteristics in individuals with ASD. In their study, Hammond et al. [20] observed an unusual asymmetry in the supraorbital and periorbital regions located anterior to the frontal cerebral pole, with a dominance towards the right side. Following this, Boutrus et al. [19] conducted a study that replicated the previously mentioned model. The findings revealed a higher depth-wise FA in individuals with ASD compared to TD children of the same age, as well as their non-ASD siblings.

The examination of facial asymmetry could potentially enhance our comprehension of the neurodevelopmental mechanisms underlying ASD and may serve as an additional biomarker for diagnostic purposes. In this sense, similar concerns occur regarding the limitations that have been outlined with regard to the increased intercanthal distance. It is noteworthy to mention that, based on the study conducted by Boutrus et al. [19], there may be an association between facial asymmetry and the severity of symptoms observed in individuals with ASD.

#### 4.1.3. Facial Masculinity

The study by Tan et al. [22] is the only one to investigate increased facial masculinity in ASD boys and children compared with TD controls. In this study, several facial variables were evaluated using 3D imaging technology and an increase in facial masculinity was observed for both sexes. Moreover, thanks to the assessment carried out by ADOS-G, facial masculinity was correlated with greater socio-communicative difficulties. For a greater understanding of the concept of facial masculinity, the two phases of the research presented are reported below. In this research, a normative sample was first analysed to determine a typical facial masculinity/femininity. In this way, a gender score was created to assess the degree of facial masculinity. Subsequently, these gender scores based on 3D images were used for a group of autistic boys and control boys and a group of autistic girls and control girls. 

#### 4.1.4. Subgroups and Clusters

Another significant finding pertains to the possibility of identifying subcategories within the ASD population by analysing cranio-facial characteristics. The presence of subgroups or clusters has been identified in a partially overlapping population through studies conducted by Aldridge et al. [18] and Obafemi-Ajayi et al. [21].

Aldridge et al. [18] delineated two distinct subgroups. Subgroup 1 exhibited a reduction in the height of the facial midline, accompanied by an increase in the breadth of the mouth, as well as the length and height of the chin. Also, subgroup 2 exhibited an augmented breadth of the upface along with a decreased height of the philtrum. In their study, Obafemi-Ajayi et al. [21] defined three distinct clusters. Cluster 1 showed a general reduction in surface facial heights, accompanied by a broader maxillary midface from the temporal landmark to the lower nose landmarks. Cluster 2 exhibited an overall increase in surface facial heights, a decrease in mid-face height, and the most extensive mouth widths. Cluster 3 displayed characteristics that lie in between those observed in cluster 1 and cluster 2.

### 4.2. Could Cranio-Facial Characteristics Predict Behavioural Features in ASD Individuals?

The inclusion of research that has included clinical assessments of ASD in examined persons is one of the review’s strengths. In six out of seven studies [18,19,20,21,22,24], the clinical diagnosis was made using the DSM criteria along with the administration of standardised tests and questionnaires that helped describe the studied population’s clinical and behavioural characteristics. Important conclusions on the predictive usefulness of cranio-facial abnormalities on autistic behaviour and the possibility to employ these characteristics for a better phenotypic and behavioural characterisation emerged from this.

In the research by Tripi et al. [24], increased intercanthal distance and reduced height of the facial midline were shown to be associated with a worse severity of ASD symptoms. These results were assessed using the ADI-R and CARS. According to Boutrus et al.’s [19] analysis based on the use of ADOS-2, facial asymmetry was also related to a more severe autistic condition. Based on the Social Affect score generated from the ADOS-G, Tan et al. [22] discovered a link between facial masculinity and higher social communication challenges.

Between the subgroups and clusters, Aldridge et al. [18] and Obafemi-Ajayi et al. [21] discovered significant differences in clinical presentation. According to Aldridge et al. [18], subgroup 1 had lower IQ scores than subgroup 2, appeared to be more severely autistic, and had higher lifetime SCQ scores. In general, subgroup 1 showed different features that were predictive of a poor outcome, including a higher risk for seizures and a higher incidence of language regression at the onset of ASD, while subgroup 2 looked to be more consistent with an Asperger syndrome diagnosis. Similarly, Obafemi-Ajayi et al.’s [21] cluster subdivision research led to the identification of a “severe autism subgroup” (cluster 2) that shared traits with subgroup 1 of Aldridge et al.’s [18] research, including severe ADI-R scores, low cognitive and functional IQ scores, the highest maternal Social Responsiveness Scale (SRS) scores, and significant language regression.

### 4.3. How Does Facial Phenotype Contribute to Our Understanding of Brain Development in ASD?

Research on the cranio-facial characteristics observed in individuals with ASD does not, by itself, explain how the brain develops or the etiological, anatomical, or functional implications of the disorder. On the other hand, data on cranio-facial abnormalities may considerably aid in a better understanding of the neurodevelopmental basis for ASD when combined with those of other research. Although the purpose of the analyses was not to provide causal explanations for the findings, the authors suggested valuable hypotheses based on the scientific literature.

As stated by Hammond et al. [20], variations in foetal and embryonic development, as well as the combination of genetics and natural selection, are likely causes of variations in face morphology. Two subgroups have been identified in the study conducted by Aldridge et al. [18]. The frontonasal process (FNP) and the midline mandibular prominences (MAND) are the main alterations of facial regions in subgroup 1. Subgroup 2 shows alterations of facial regions that develop from embryonic FNP. Thus, according to Aldridge et al. [18], developmental alterations in the face and brain may be associated with changes in the expression of genes involved in the development of these regions (such as FGF8, SHH, BMP2, and WNT). In accordance with Hammond et al. [20], an indirect consequence of asymmetric brain development on facial development may be responsible for the prominent right brain asymmetry of the frontal pole in males with ASD. Genetic factors acting simultaneously on face and brain development in ASD is an alternate hypothesis that is not necessarily mutually exclusive.

In addition to the elements mentioned by Hammond et al. [20], Boutrus et al. [19] investigated facial asymmetry and recommended delving into the caudate nuclei. In ASD studies, it was shown that ASD individuals had significantly larger caudate nuclei than corresponding TD participants, especially the right nucleus. The examination of caudate nuclei and FA in diagnosed individuals could help to distinguish clinically and neurobiologically distinct subgroups in ASD due to its asymmetric presentation and association with confined and repetitive behavioural symptomatology.

According to the Tan et al. [22] study, prenatal testosterone may contribute to the development of autistic-related behaviours and facial characteristics. In fact, there is an association between prenatal testosterone and the masculinisation of the face, which in turn seems to be related to ASD.

Another study by Topal et al. [23] revealed that males had significantly greater orbital measures than females. A study of healthy individuals revealed that all diameters of the orbital soft tissues are bigger in men than in women. Topal et al. [23] claim that these major measurements in ASD males may support the idea that autism is an “extremely masculine brain syndrome”.

Based on anthropometric and MRI investigations, the studies of Tripi et al. and Topal et al. [23,24] both explored and found evidence of an increase in the distance between orbits among individuals with ASD. The study conducted by Cheung et al. [29], which discovered an association between the greater distance between orbits and an early growing head size among individuals with ASD, was mentioned by both research groups. Both study groups found a correlation between the interorbital distance and the volume of grey matter in the amygdala, which extended bilaterally to ventro-medial fusion with the head of the hippocampus and the lower pole of the upper temporal lobes.

Additionally, Tripi et al. [24] provided a study showing increased amygdala in preschoolers who eventually received an ASD diagnosis. The association between the expansion of the amygdala and the widening of the eye sockets may be a sign of early amygdala-related brain dysfunction. Amygdala dysfunction in autism spectrum disorders may be related to “social brain” dysmorphology, as it is frequently involved in emotion regulation, theory of mind, and gaze perception. These findings support the idea that the development of “social brain” structures (such as the amygdala) and the optical differentiation of neural crest cells are closely interconnected. A decrease in the height of the midline of the face is also a sign of abnormalities with the midline structures of the human embryonic face, according to Tripi et al. [24] Since the embryonic notochord is a median structure, abnormal brain expansion throughout foetal life may compromise midline cranio-facial development. Thus, an early anteroposterior expansion of the neurocranium may account for the increased interorbital distance in those with autism spectrum disorders.

### 4.4. Strengths and Limitations

Some of the strengths and limitations of this review have been addressed in the discussion. The use of the PRISMA statement method and the evaluation of quality using the QuADS tool are certainly strengths of this review. In addition, the inclusion of studies that have all included a quantitative and instrumental evaluation of cranio-facial features and a clinical evaluation of autistic symptomatology significantly reinforces the considerations that may derive from the studies examined. Similarly, the exclusion of articles concerning specific genetic conditions or prenatal exposure to alcohol may have increased the specificity of the research, allowing important biases due to the characteristics of these conditions to be excluded. This review also encountered several limitations. It is relevant to underline how most of the research in this field is based on samples of Caucasic ethnicity, and therefore there is scarce evidence about other ethnicities that might benefit from further studies. In the same way, even if a part of the studies included in this review provided samples of ASD females, the studies had significantly larger ASD males’ samples, reflecting a difference in the epidemiological distribution of the disorder but still evidencing the need for a deepening of research on autistic females. The use of different methodologies also presents important considerations. In two studies, photogrammetry is used [23,24], while in the other five studies, 3D imaging is used [18,19,20,21,22]. The first method, photogrammetry, has clear advantages that include lower cost and greater ease of use, while 3D imaging in the face of greater image accuracy poses considerable difficulties regarding a considerable economic cost and the need for greater compliance of the patients involved.

This review provides vital insights into the potential correlation between facial dysmorphism and autism. Nevertheless, it is crucial to note the limitations of this study and highlight the need for more extensive research efforts that explore the nuanced complexities of this correlation. It is crucial to thoroughly investigate and identify possible factors that may influence the relationship between facial dysmorphism and autism, such as intellectual impairment, neurological conditions, and mental comorbidities. This will help us enhance our comprehension of the intricate interaction between these elements. Further research, taking into account these variables, will be crucial in progressing this field and developing a more detailed understanding of the fundamental systems that control the supposed connection between facial dysmorphism and autism spectrum disorders.

## 5. Conclusions

The analysis of facial abnormalities could be a potential candidate diagnostic biomarker that may help in the early detection of ASD. Based on anthropometric and instrumental measures, it may be possible to identify characteristics in certain individuals with ASD that may be predictive of the severity of autistic symptoms. A phenotype that has a longer intercanthal distance and a shorter facial midline seems to be associated with more severe ASD symptoms. Facial masculinity and asymmetry might both be considered reliable predictive biomarkers of a worse symptomatologic presentation. Further research in genetics, epigenetics, and imaging techniques is necessary to better understand the processes of neurodevelopment in ASD by studying cranio-facial abnormalities.

## Figures and Tables

**Table 1:** Characteristics of the seven studies included by the researchers.

Reference Article (Reference)	Title	Publication Year	Country	Study Design	QuADS Tool—Quality Rating
Hammond, P. [20]	Face-brain asymmetry in autism spectrum disorders	2008	Canada	Case–control	64.1%
Aldridge, K. [18]	Facial phenotypes in subgroups of prepubertal boys with autism spectrum disorders are correlated with clinical phenotypes	2011	United States of America	Case–control	71.8%
Obafemi-Ajayi, T. [21]	Facial structure analysis separates autism spectrum disorders into meaningful clinical subgroups	2015	United States of America	Case–control	71.8%
Tan, D.W. [22]	Hypermasculinised facial morphology in boys and girls with autism spectrum disorder and its association with symptomatology	2017	Australia	Case–control	69.2%
Tripi, G. [24]	Cranio-facial characteristic in children with autism spectrum disorders (ASD)	2019	France	Cross-sectional	79.5%
Boutrus, M. [19]	Increased facial asymmetry in autism spectrum conditions is associated with symptom presentation	2019	Australia	Case–control	87.2%
Topal, Z. [23]	Anthropometric analysis of the orbital region in children with autism spectrum disorder and healthy controls	2021	Turkey	Case–control	41.0%

**Table 2:** Relevant information gathered from each included study.

Reference Article (Reference)	Purpose of the Study	Participants	Age Range	Tools for Autism Evaluation	Methods of Evaluation of Facial Phenotype	Facial Phenotype Evaluation	Main Findings
Hammond, P. [20]	To compare facial morphology of ASD boys and first-degree relatives to that of unrelated controls	- ASD boys (Case): 72 - Unrelated unaffected male controls (Control): 105 - Caucasian ethnicity - Identifiable syndromes excluded	- Case: 2.2–18.2 years old - Control: 2.0–18.0 years old	- Clinical evaluation - ADI-R - ADOS-G	3D imaging 3dMD System	Asymmetry index of: - Face - Supra-orbit - Periorbit - Perinasal - Perioral	- Faces of ASD boys have atypical right dominant asymmetry of the supraorbital and periorbital regions anterior to the frontal cerebral pole
Aldridge, K. [18]	To investigate if children with ASD display a subtle but distinct facial phenotype compared to TD children. If there are subgroups of facial phenotypes within the ASD group.	- ASD boys (Case): 64 - Typically developing boys (Control): 41 - Caucasian ethnicity - Identifiable syndromes excluded	- Case: 8–12 years old - Control: 8–12 years old	- Clinical evaluation - ADI-R - ADOS (for 36 out of 64 ASD boys)	3D imaging 3dMD System	Subgroups based on: - Euclidean Distance Matrix Analysis - Principal Coordinates Analysis	- Statistically significant facial morphology differences compared to TD: increased mouth, orbits and upface breadth, flattened nasal bridge, and reduced philtrum and maxillary height. Two clinically distinct subgroups were found. - Subgroup 1: decreased facial midline height, increased mouth breadth, and chin length and height. - Subgroup 2: increased upper face breadth and decreased philtrum height. The subgroups differ biologically and etiologically. With higher SCQ life-time scores, subgroup 1 appears more severely autistic than subgroup 2. IQ appears lower in subgroup 1. Subgroup 1 had a higher risk of seizures and language regression at ASD onset, indicating poor outcomes. Subgroup 2 resembles Asperger syndrome.
Obafemi-Ajayi, T. [21]	To determine whether facial morphology constitutes viable biomarker for delineation of discrete autism spectrum disorders (subgroups)	- Prepuberal boys with essential autism (Case): 62 - Typically developing prepubertal boys (Control): 36 - Caucasian ethnicity - Identifiable syndromes excluded	- Case: 8–12 years old - Control: 8–12 years old	- Clinical evaluation - ADI-R - ADOS (for 42 out of 62 ASD boys)	3D imaging 3dMD System	Clusters based on: - Facial height - Mid face height - Mid face breadth - Lower face height - Mouth width	- Cluster 1: lower surface facial heights and a broader maxillary midface from temporal to lower nose landmarks. - Cluster 2: higher surface facial heights, lower mid-face height, longer mouth widths. - Cluster 3 is between clusters 1 and 2. - Cluster 2 is characterised by severe ADI-R scores, low cognitive and functional IQ scores, highest maternal SRS scores, and significant language regression (similar to Aldridge et al. [18] subgroup 1)
Tan, D.W. [22]	To investigate whether prepubescent boys and girls with ASD present increased facial masculinity compared to TD controls	- ASD boys (Case): 54 - TD boys (Control): 54 - ASD girls (Case): 20 - TD girls (Control): 60 - Caucasian ethnicity	- Boys: 3.01–12.52 years old - Girls: 4.24–11.78 years old	- Clinical evaluation - ADOS-G	3D imaging 3dMD System	Facial variables: - Facial area - Gender scores - Alar-base width - Nose height - Upper lip height - Outer-canthal width - Forehead height - Nose height	- For each sex, increased facial masculinity was observed in the ASD group relative to control group. Facial masculinity in the ASD group correlated with more social-communication difficulties based on the Social Affect score derived from the ADOS-G
Tripi, G. [24]	To investigate the topographical pattern of cranio-facials anomalies in ASD children, and to determine whether these anomalies would correlate with intensity of ASD symptoms and overall functioning	- Prepuberal boys with ASD: 28 - Prepuberal girls with ASD: 5 - Caucasian ethnicity - Identifiable syndromes excluded	4–12 years old	- Clinical evaluation - ADI-R - CARS - WAIS III	Photogrammetry FlashCAD and classical anthroposcopy	Cranio-Facial Indexes: - Cephalix index - Facial index - Intercanthal index - Nasal index; - Facial-mouth width index	- Two craniofacial markers were significantly correlated with autism severity: increased orbital hyperthelorism and decrease of height of the facial midline - Dolicocephalic head shape not correlated with autism severity
Boutrus, M. [19]	To investigate the replicability of increased facial asymmetry in autistic children compared to non-autistic peers	- ASC boys: 58 - TD boys: 72 - ASC girls: 14 - TD girls: 38 - Full siblings: 38 (22 M, 16 F) - Caucasian ethnicity - Identifiable syndromes excluded	- ASC mean age: 9.36 - TD mean age: 8.98 - Siblings mean age: 7.25	- Clinical evaluation - ADOS-2	3D imaging 3dMD System	- Horizontal Facial Asymmetry - Vertical Facial Asymmetry - Depth Facial Asymmetry	Autistic individuals have increased depth-wise FA compared to age-matched TD children as well as full siblings without ASC. Relationship between FA and ASC symptom severity
Topal, Z. [23]	To examine the orbital region in children with ASD and comparison with the controls	- ASD boys (Case): 82 - TD boys (Control): 53 - ASD girls (Case): 19 - TD girls (Control): 41	- Case average age: 8.02 ± 3.41 - Control average age: 9.09 ± 4.57	- Clinical evaluation	Photogrammetry ImageJ (version 1.50b)	- Outer canthal distance - Palpebral fissure distance or intercanthal distance - Inner canthal distances (bilateral) - Interpupillary distance	Results of this study show that orbital distances are increased in autistic males, but there is no difference in females compared to control group

## Data Availability

Not applicable.
